# Well begun is half done

**DOI:** 10.1007/s12471-016-0908-5

**Published:** 2016-10-21

**Authors:** R. Pisters, M. de Booij, O. Reuchlin, S. Rasoul

**Affiliations:** 1 Department of Cardiology, Zuyderland MC Heerlen, Heerlen, The Netherlands; 2 Department of Radiology, Zuyderland MC Heerlen, Heerlen, The Netherlands

**Fig. 1:**
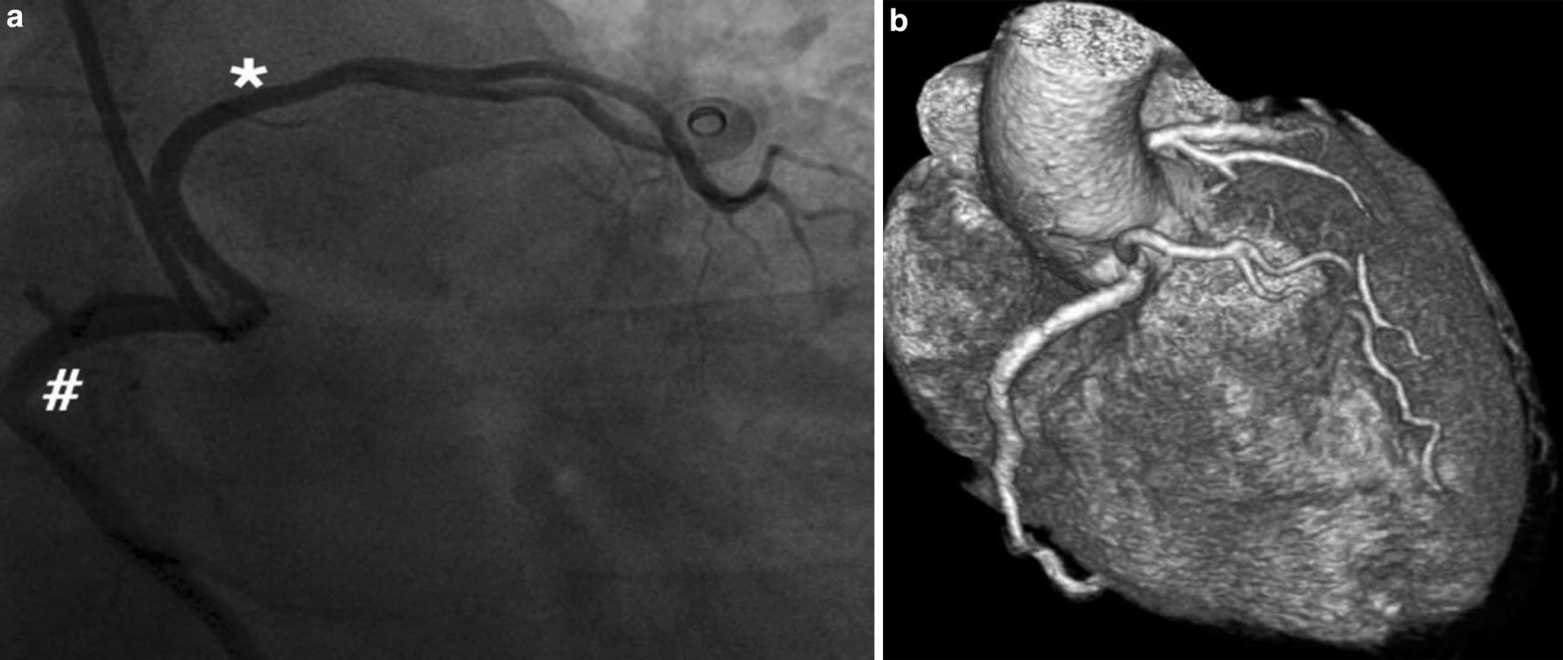
**a** Coronary angiography displaying opacification of the right coronary cusp of the right coronary artery (#) and the anomalous origin of the left anterior descending coronary artery (*). **b** Coronary computed tomography revealed no evidence of external compression

Chest pain and palpitations caused a 57-year-old female with Hashimoto disease to visit our outpatient clinic. Physical examination, laboratory testing and ECG showed no abnormalities. Given the 13-beat non-sustained ventricular tachycardia on Holter monitoring and persistent chest pain, cardiac catheterisation was performed. This showed the origin of the left anterior descending artery (LAD) to be anomalous, coming from the right coronary cusp (Fig. 1a). Coronary computed tomography showed no overt external compression (Fig. 1b).

Coronary artery anomalies are an uncommon finding during life (1.3 %), particularly anomalies of the LAD (0.017 %) [1]. They are predominantly of an asymptomatic, benign nature and rarely compromise haemodynamics or cause sudden cardiac death. However, interarterial and especially intramural aortic course increases the likelihood of this happening [2, 3]. Considering the potential significance and implications, clinical awareness and angiographic recognition of coronary anomalies is critical [4]. Here, the LAD anomaly was an isolated phenomenon without an interarterial course.
